# Recent progress in efficient hybrid lead halide perovskite solar cells

**DOI:** 10.1088/1468-6996/16/3/036004

**Published:** 2015-06-18

**Authors:** Jin Cui, Huailiang Yuan, Junpeng Li, Xiaobao Xu, Yan Shen, Hong Lin, Mingkui Wang

**Affiliations:** 1 Michael Grätzel Center for Mesoscopic Solar Cells, Wuhan National Laboratory for Optoelectronics, Huazhong University of Science and Technology, Wuhan, Hubei 430074, People’s Republic of China; 2 State Key Laboratory of Advanced Technologies for Comprehensive Utilization of Platinum Metals, Kunming Institute of Precious Metals, Kunming 650106, People’s Republic of China; 3 School of Materials Science and Engineering, Tsinghua University, Beijing, 100084, People’s Republic of China

**Keywords:** perovskite, solar cell, power conversion efficiency, surface modification, interface

## Abstract

The efficiency of perovskite solar cells (PSCs) has been improved from 9.7 to 19.3%, with the highest value of 20.1% achieved in 2014. Such a high photovoltaic performance can be attributed to optically high absorption characteristics and balanced charge transport properties with long diffusion lengths of the hybrid lead halide perovskite materials. In this review, some fundamental details of hybrid lead iodide perovskite materials, various fabrication techniques and device structures are described, aiming for a better understanding of these materials and thus highly efficient PSC devices. In addition, some advantages and open issues are discussed here to outline the prospects and challenges of using perovskites in commercial photovoltaic devices.

## 1. Introduction

Solar energy is the most abundant energy resource on Earth, where every minute enough sunlight reaches the Earth’s surface to meet the world’s energy demands for one year. Currently the conversion of sunlight into energy can be achieved through solar photovoltaic (PV) cells, concentrated PVs and solar thermal technologies. Today, solar energy provides only a small fraction of total global electricity generation (∼1%) but the use of solar PVs is expanding rapidly due to the annual decrease in the cost of such technologies [1]. Solar cells made of crystalline silicon are often referred as first generation solar cells and have dominated the PV market over the past half century [2, 3]. ‘Third generation’ PV technologies have been developed to pursue high power conversion efficiency (PCE) and low cost, these include light-condensed cells, organic PVs (OPVs), dye-sensitized solar cells (DSSCs), organic–inorganic hybrid solar cells, and so on. The large flexibility in the shape, color and transparency of third generation solar cells, particularly DSSCs, makes them one of the most promising technologies for photo-to-electricity conversion applications [4–8]. Currently, in the laboratory, DSSCs have reached 13% efficiency under standard reporting conditions [9], providing a credible alternative to the conventional p–n junction PV devices. The major disadvantage in DSSC design is the use of a liquid electrolyte, which suffers from temperature stability problems. Replacing the liquid electrolyte with a solid one is a major, ongoing field of research. Not only the stability, but also the open circuit voltage of a DSSC could be expected to improve when a solid-state hole transfer material (HTM) is used. Thus far, 2, 2’,7,7’-tetrakis(N,N-di-p-methoxyphenylamine)-9,9’-spirobifluorene (spiro-OMeTAD) and its derivatives have been the most popular HTMs in solid-state DSSCs [10–12]. They were first introduced by Bach *et al* in 1998 [13]. However, a satisfactory PV performance for solid-state cells containing such materials can only be achieved with thin mesoporous photoanodes because of the difficulty of infiltration of HTMs [14, 15]. Thus, only light absorbers with high extinction coefficients or a wide absorption spectrum are able to provide sufficient sunlight harvesting in thin films [16–18].

Hybrid lead iodide perovskite materials exhibit a high absorption coefficient up to 1.5 × 10^4^ cm^−1^ at 550 nm, estimated from a CH_3_NH_3_PbI_3_ coated nanocrystalline TiO_2_ thin film, which indicates that the penetration depth for 550 nm light is only 0.66 *μ*m [19]. In 2009, Kojima *et al* reported the first study of the organic lead halide compounds CH_3_NH_3_PbBr_3_ and CH_3_NH_3_PbI_3_ as sensitizers in photo-electrochemical cells [20]. They measured a moderate PCE of 3.81% for the CH_3_NH_3_PbBr_3_-based device and 3.13% for the CH_3_NH_3_PbBr_3_-based device. The stability of these devices was poor in a liquid electrolyte cell configuration. However, the high absorption coefficient makes them one of the best choices for solid-state solar cells. In mid-2012, solid-state DSSCs based on CH_3_NH_3_PbI_3_ demonstrated a PCE of 9.7%, in which spiro-OMeTAD was used as the HTM [21]. The devices exhibited a large photocurrent (*J*_SC_) enhancement exceeding 17 mA cm^−2^, which was superior to the best conventional solid-state DSSCs with organic light-harvesting materials. The use of a solid-state HTM dramatically improved the device’s stability compared to CH_3_NH_3_PbI_3_-sensitized liquid junction cells, although this remains the main issue in the development of perovskite solar cell (PSC). Since then, the PCEs of PSCs have experienced a burst of development. It is worth mentioning that a new method for the fabrication of CH_3_NH_3_PbI_3_-based PSCs with 15.0% efficiency was introduced by Burschka *et al*, in which the PSCs are sequentially deposited from separate solutions of CH_3_NH_3_I and PbI_2_ [22]. Planar junction PSCs have also been developed by Liu *et al* and high efficiency devices have been achieved using a dual-source vapor deposition (DSVD) method [23]. Very recently, a certified PCE record of ∼20.1% was attained through interfacial modification of the perovskite layer and electrodes as well as film preparation [24].

PSCs can adopt the merits of inorganic materials, such as stability, high carrier mobility and a compatible fabricating process, and utilize the advantages of organics, such as tunable light absorption, adjustable molecular structures for energy band alignment and facile solution processability. This review descibes the basic characteristics of hybrid lead iodide perovskite materials, various fabrication techniques and device structures to provide better understanding and insight into high-performing PSCs. In addition, the most notable problems and recent progress in research are highlighted with regard to further improvement of PSCs.

## 2. The basics of hybrid lead halide perovskites

Perovskite is the name of a crystal structure, first found in the inorganic mineral CaTiO_3_ (ABX_3_), with a cubic unit cell. In the unit cell, the A-cation resides at the eight corners of the cube surrounded by twelve X-anions, while the B-cation is located at the body center which is surrounded by six X-anions (located at the face centers) in an octahedral [BX_6_]^4−^ cluster [20, 25]. The A and B sites can accommodate inorganic cations of various valences and ionic radii. Alternatively, small organic cations such as CH_3_NH_3_^+^, C_2_H_5_NH_3_^+^ and HC(NH_2_)_2_^+^ can be used to replace inorganic cation A and create organic–inorganic hybrid materials [26, 27]. The metal cations (B) are typically divalent metal ions such as Pb^2+^, Sn^2+^ or Cu^2+^, and the X anions are halides (Cl^−^, Br^−^, I^−^) [25, 28–30].

**Figure 1.**
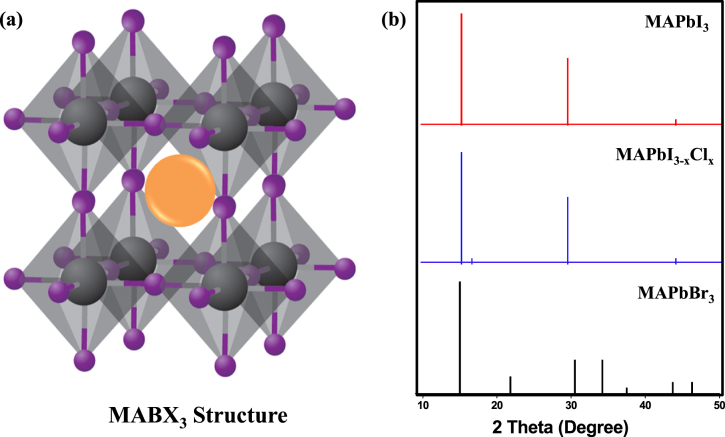
Crystal structure of MABX_3_ (a) and powder XRD pattern (b) (peaks at 2*θ* angles of 14.1, 28.4 and 43.1 for MAPbI_3_) of Pb-based halide perovskite materials. The metal cation (B, grey sphere) is located at the body center of the cubic composed of six halide X-anions (purple). The orange sphere represents the CH_3_NH_3_^+^ ion (MA cation). The XRD values are taken from [54] and [41].

Weber reported the replacement of A by methylammonium cations (MA=CH_3_NH_3_^+^) to generate the first three-dimensional organic–inorganic hybrid perovskites [31, 32]. figure 1(a) shows the general structure of these organometal halide CH_3_NH_3_BX_3_ (B=Pb or Sn, X=Cl, Br or I) materials. Methylammonium lead iodide, CH_3_NH_3_PbI_3_, has interesting optical and electronic properties that have been actively investigated during the past two decades [33, 34]. CH_3_NH_3_PbI_3_ is a semi-conducting pigment with a direct bandgap of 1.55 eV corresponding to an absorption onset of 800 nm, which makes this material a good light absorber over the whole visible solar emission spectrum [25, 33]. The photon-generated excitons in CH_3_NH_3_PbI_3_ have a weak binding energy of less than 50 meV [35, 36], which means that most of them can be dissociated very rapidly into free carriers at room temperature [37]. The electrons and holes produced in this material exhibit small effective masses resulting in high carrier mobilities ranging from 24 ± 7 cm^2^ V^−1^ s^−1^ for electrons to 105 ± 35 cm^2^ V^−1^ s^−1^ for holes [38]. Their recombination occurs on a timescale of hundreds of nanoseconds, resulting in long carrier-diffusion lengths—that is, the average distance that can be covered by carriers before they recombine—ranging between 100 nm and 1000 nm (recently, electron–hole diffusion lengths of >175 *μ*m in MAPbI_3_ single crystals have also been reported) [38–40]. Even though these appealing properties had already been known for more than 20 years, the extraordinary potential of hybrid perovskites in PV applications was only revealed five years ago [19, 20].

**Figure 2.**
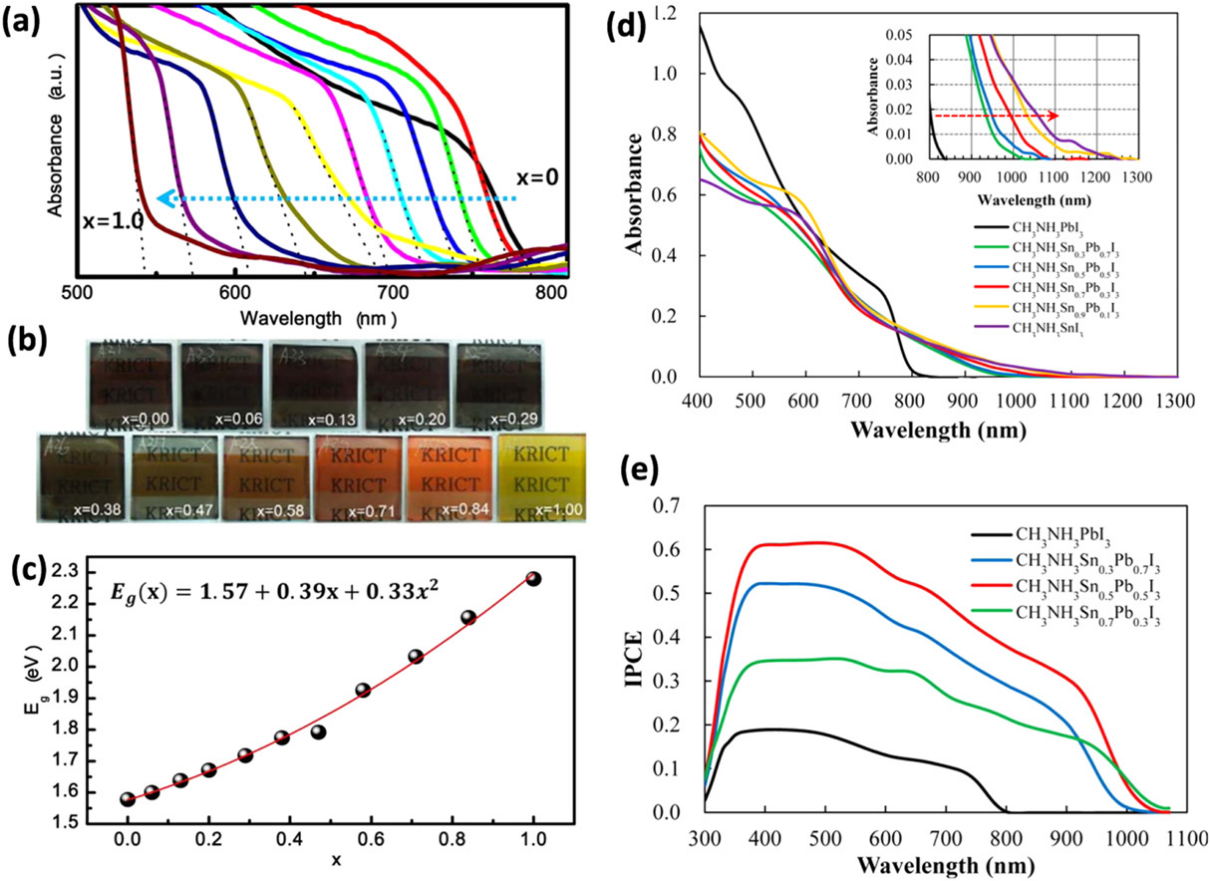
Photographs and UV–vis absorption spectra of MAPb(I_1−*x*_Br_*x*_)_3_. (a) UV–vis absorption spectra of mp-TiO_2_/MAPb(I_1−*x*_Br_*x*_)_3_ films measured using an integrating sphere. (b) Photographs of three-dimensional TiO_2_/MAPb(I_1−*x*_Br_*x*_)_3_ bilayer nanocomposites on a FTO glass substrates. (c) A quadratic relationship of the bandgaps of MAPb(I_1−*x*_Br_*x*_)_3_ as a function of Br composition (*x*). Reprinted with permission from [42]. Copyright 2013 American Chemical Society. (d) Electronic absorption spectra of MASn_*x*_Pb(_1−*x*_)I_3_ perovskite coated on porous TiO_2_ with (e) corresponding incident photon-to-current conversion efficiency curves for MASn_*x*_Pb(_1−*x*_)I_3_ perovskite/poly(3-hexylthiophene) solar cells. Reprinted with permission from [47]. Copyright 2014 American Chemical Society.

**Figure 3.**
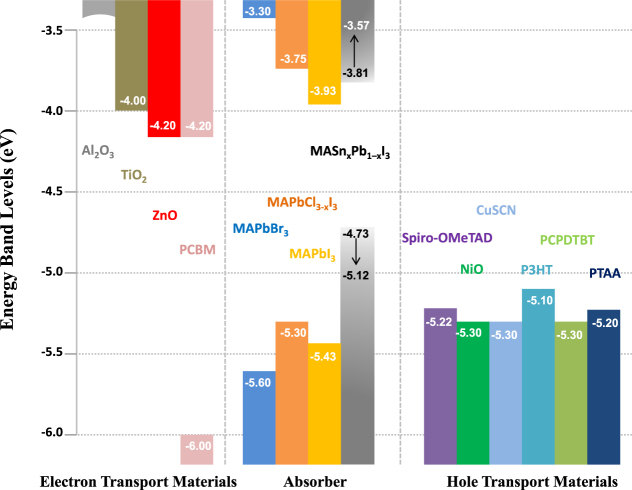
Energy level diagram of the materials used in PSCs.

Iodine can be partially or entirely replaced by other halogens (Br and Cl) to form different perovskite materials, among which CH_3_NH_3_PbBr_3_ and CH_3_NH_3_PbI_3−*x*_Cl_*x*_ have attracted intense attention. X-ray diffraction (XRD) patterns of these Pb-based halide perovskite materials are shown in figure 1(b). We note that it is not certain whether the chemical formula of CH_3_NH_3_PbI_3−*x*_Cl_*x*_ is suitable or not, as the role of chlorine in these materials is still under debate. Interestingly, the hybrid lead halide perovskite incorporates two halides (e.g., iodide with bromide), allowing the continuous tuning of the optical bandgap to cover the most visible spectrum (figures 2(a)–(c)). The introduction of Br elevates the conduction band (CB) and lowers the valence band (VB) for CH_3_NH_3_PbBr_3_, whose direct bandgap is ∼2.2 eV (figure 3) [43–46]. Raising the CB minimum is favorable for energy band matching between TiO_2_ and CH_3_NH_3_PbBr_3_; it suppresses geminate recombination, which results in an improvement of the open-circuit voltage (*V*_OC_). Unfortunately, the large bandgap of CH_3_NH_3_PbBr_3_ limits light absorption with an onset wavelength less than ∼550 nm, which would subsequently greatly cut down the photocurrent. Photon-generated excitons of CH_3_NH_3_PbBr_3_ have a higher binding energy of about 150 meV compared to CH_3_NH_3_PbI_3_ (≤50 meV) [36, 44]. Therefore, the PCE of PSCs using CH_3_NH_3_PbBr_3_ is still lower than that of CH_3_NH_3_PbI_3_. By partially substituting Pb with Sn one can continuously tune the optical bandgap [47]. The reported threshold of the incident photon to current efficiency of their devices was extended to 1060 nm (figures 2(d) and (e)).

Different to Br, the introduction of Cl was believed to elevate the formation of perovskite grains by decreasing the surface or bulk defects [25, 46]. This enhanced crystallinity can accelerate the transfer and diffusion of photon-generated carriers, thus increasing the electron–hole diffusion lengths. It was reported that the diffusion length of CH_3_NH_3_PbI_3−*x*_Cl_*x*_ exceeds 1 *μ*m, which is far longer than that for CH_3_NH_3_PbI_3_ (∼100 nm) [40].

Regarding the optoelectronic properties of hybrid lead halide perovskite materials, the presence of defect sites in these materials has thus far been ignored, in contrast to c-Si and other crystalline semiconductors, where minimizing impurities and crystal imperfections is critical to prepare the highest efficiency PV cells [48, 49]. A high density of point defects (i.e., Schottky defects and Frenkel defects) was created while tuning the ratio of CH_3_NH_3_I and PbX sources during the synthesis procedure. Their properties directly determine the charge carrier diffusion length. However, the calculation of the Schottky defects [50] such as PbI_2_ and CH_3_NH_3_I vacancies shows no formation of gap states within the bandgap [51]. These results indicate that point defects in the bulk do not act as charge recombination centers. It was further discovered that the formation energy for deep defects (iodine interstitial in *β*-CH_3_NH_3_PbI_3_, tetragonal room-temperature phase) can be further increased by Cl doping [52].

## 3. Perovskite solar cell device structure

**Figure 4.**
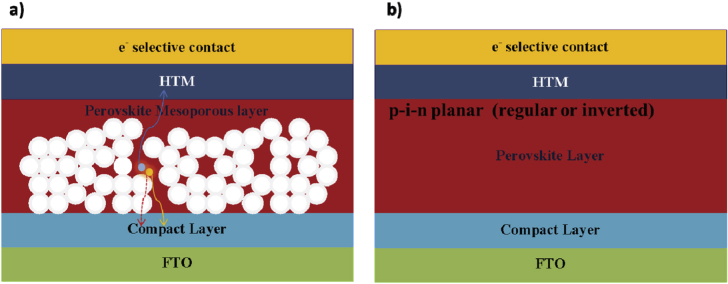
The structure of PSCs. (a) The model structure of mesoporous halide PSCs and (b) the model structure of planar heterojunction-structure halide PSCs.

During 2014 there were several demonstrations of cells with efficiencies >16% [53, 54], along with predictions of a ‘straightforward path to well above 20%’. A range of device strategies yielding high efficiencies has been reported: the regular [22] and inverted electrical geometries [55], with [56, 57] or without mesoporous oxide supports [23, 55] and using a variety of transport materials (depicted in figure 3) [53, 58–61]. Device performance with various PSC structures is summarized in table 1. Figure 4 shows the schematic structures and energetics of two typical devices: mesoscopic nano-structured and planar structured PSC devices. Charge separation by electron injection from the perovskite to the TiO_2_ is obvious in cases where perovskite dots are adsorbed onto the TiO_2_ surface. [21] In this case, the working principle is expected to be similar to DSSCs. However, Lee *et al* reported a PSC comprised of mesoporous Al_2_O_3_ instead of TiO_2_ [65], demonstrating that the Al_2_O_3_ merely acted as a scaffold layer without injection of photo-excited electrons. Photo-induced absorption spectroscopy measurements revealed that hole transfer was highly effective from the photo-excited perovskite to the spiro-OMeTAD, generating long-lived charge species within the perovskite layer coated on the Al_2_O_3_ nanoparticles. Furthermore, small-perturbation transient photocurrent decay measurements revealed that charge collection was ten times faster in the Al_2_O_3_-based cell than in the TiO_2_-based one, indicating much faster electron diffusion through the perovskite phase than in the n-type TiO_2_. Sum and Snaith further investigated the balanced long-range electron–hole diffusion properties of organometallic perovskite materials [39, 40]. The electron and hole diffusion lengths were estimated to be ∼130 nm and ∼100 nm, respectively, for samples prepared from a CH_3_NH_3_I + PbI_2_ mixture, while longer diffusion lengths of ∼1069 nm for electrons and ∼1213 nm for holes were measured for samples prepared using a 3CH_3_NH_3_I + PbCl_2_ mixture. Subsequent work using iodide prepared by the vapor-phase CH_3_NH_3_I conversion of PbI_2_ resulted in good carrier collection in 350 nm thick iodide films, suggesting that the diffusion lengths exceed this thickness [66]. This is supported by recent electron-beam measurements [67]. Electron beam-induced current (EBIC) imaging studies of CH_3_NH_3_PbI_3_ layers prepared from PbI_2_ and PbCl_2_ sources revealed that the charge diffusion lengths were about 1 *μ*m for both samples, in which the diffusion length for holes was longer than that for electrons for the case of CH_3_NH_3_PbI_3_ prepared without a chloride source.

**Table 1.** Compilation of high efficiency PSCs. States: c = compact, mp = mesoporous and MA=CH_3_NH_3_.

	Device structure	Active area	Device parameters	Efficiency	References/Year
Regular structure	**With scaffold layer**				
			*J*_SC_ = 20.0 mA cm^–2^		
	FTO/c-TiO_2_/mp-TiO_2_/MAPbI_3_/spiro-OMeTAD/Au	0.285 cm^2^	*V*_OC_ = 0.99 V, FF = 0.73	15.0%	[22] 2013
			*J*_SC_ = 21.2 mA cm^–2^		
	FTO/c-TiO_2_/mp-TiO_2_/MAPbI_3_/spiro-OMeTAD/Au	0.096 cm^2^	*V*_OC_ = 1.02 V, FF = 0.77	16.7%	[53] 2014
			*J*_SC_ = 21.5 mA cm^–2^		
	FTO/c-TiO_2_/mp-Al_2_O_3_/MAPbI_3-x_Cl_x_/spiro-OMeTAD/Ag	0.0625 cm^2^	*V*_OC_ = 1.02 V, FF = 0.71	15.9%	[57] 2014
			*J*_SC_ = 19.5 mA cm^–2^		
	FTO/c-TiO_2_/mp-TiO_2_/MAPb(I_3-x_Br_x_)/Triarylamine(PTAA)/Au	0.094 cm^2^	*V*_OC_ = 1.09 V, FF = 0.76	16.2%	[79] 2014
	**Planar**				
			*J*_SC_ = 21.5 mA cm^–2^		
	FTO/c-TiO_2_/vapor-deposited MAPbI_3-x_Cl_x_/spiro-OMeTAD/Au	0.076 cm^2^	*V*_OC_ = 1.07 V, FF = 0.67	15.4%	[23] 2013
			*J*_SC_ = 20.4 mA cm^–2^		
	ITO/c-ZnO/MAPbI_3_/spiro-OMeTAD/Ag	0.071 cm^2^	*V*_OC_ = 1.03 V, FF = 0.75	15.7%	[58] 2013
			*J*_SC_ = 22.7 mA cm^–2^		
	FTO/c-Y:TiO_2_/spin-coated MAPbI_3-x_Cl_x_/spiro-OMeTAD/Au	0.100 cm^2^	*V*_OC_ = 1.13 V, FF = 0.75	19.3%	[54] 2014
	**HTM-free**				
			*J*_SC_ = 19.0 mA cm^–2^		
	FTO/c-TiO_2_/mp-TiO_2_/MAPbI_3_/Au	0.090 cm^2^	*V*_OC_ = 0.84 V, FF = 0.68	10.8%	[72] 2014
			*J*_SC_ = 22.8 mA cm^–2^		
	FTO/c-TiO_2_/mp-TiO_2_/mp-ZrO_2_/MAPbI_3_/C	0.070 cm^2^	*V*_OC_ = 0.85 V, FF = 0.66	12.8%	[73] 2014
Inverted structure	**With scaffold layer**				
			*J*_SC_ = 13.2 mA cm^–2^		
	ITO/c-NiO_x_/mp-NiO/MAPbI_3_/PCBM/BCP/Al	0.060 cm^2^	*V*_OC_ = 1.04 V, FF = 0.69	9.5%	[56] 2014
			*J*_SC_ = 18.0 mA cm^–2^		
	FTO/c-NiO_x_/mp-Al_2_O_3_/MAPbI_3_/PCBM/BCP/Ag	0.090 cm^2^	*V*_OC_ = 1.04 V, FF = 0.72	13.5%	[62] 2014
	**Planar**				
			*J*_SC_ = 15.2 mA cm^–2^		
	FTO/c-NiO/MAPbI_3_/PCBM/BCP/Au	0.100 cm^2^	*V*_OC_ = 1.10 V, FF = 0.59	9.8%	[63] 2014
			*J*_SC_ = 20.6 mA cm^–2^		
	ITO/PEDOT:PSS/MAPbI_3_/PCBM/Al	0.096 cm^2^	*V*_OC_ = 0.94 V, FF = 0.78	15.3%	[94] 2013
			*J*_SC_ = 19.9 mA cm^–2^		
	ITO/PEDOT:PSS/MAPbI_3-x_Cl_x_/PCBM/Ca/Au	0.100 cm^2^	*V*_OC_ = 1.05 V, FF = 0.78	16.3%	[64] 2014
			*J*_SC_ = 21.5 mA cm^–2^		
	ITO/DPP-TT/MAPbI_3_/PCBM/Au	0.200 cm^2^	*V*_OC_ = 1.05 V, FF = 0.72	16.5%	[35] 2014

**Figure 5.**
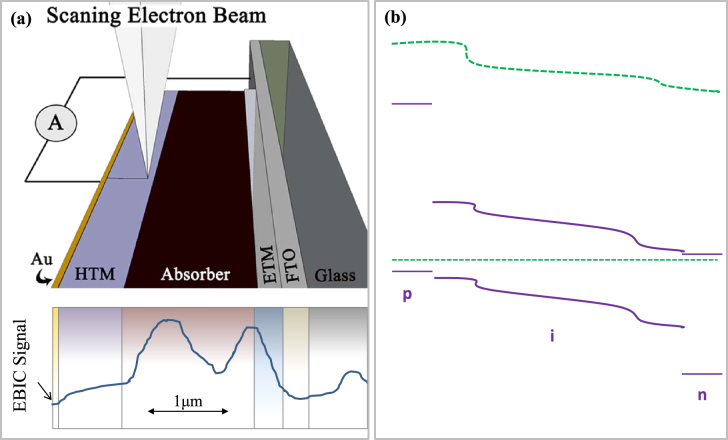
(a) Schematic of an EBIC experiment. A scanned electron beam generates a cloud of carriers, creating a position-dependent current in a short-circuiting load. Reprinted with permission from [67]. Copyright 2014 American Chemical Society. (b) A schematic band diagram of CH_3_NH_3_PbI_3−*x*_Cl_*x*_ perovskite-based cells under light in a p–i–n device structure. The diagram is not to scale and shows only the relative positions of the energy bands. The green dashed lines show the relative quasi-Fermi level.

Due to the long carrier lifetime and long diffusion length, bulk organometallic perovskite can also work efficiently without a mesoporous oxide layer or scaffold layer. Perovskite was deposited directly between two thin selective compact layers to construct planar heterojunction-structure halide PSCs. The efficiency of planar PSCs was increased up to ∼19.3% [54]. Carrier transport and recombination were compared for the mesoscopic structure and the planar structure. It was shown that the former was less efficient than the latter, this being the main factor affecting the device’s PV performance [68]. The results showed that the transport rate was the same but the recombination rate was higher for the mesoscopic structure. Recently, Edri *et al* addressed the fundamental issue of how these planar PSCs work by applying a scanning electron microscopy-based technique to cell cross-sections [69]. By mapping the variation in efficiency of charge separation and collection in the cross-sections, they showed the presence of two prime high efficiency locations: one at/near the absorber/hole-blocking-layer and the second at/near the absorber/electron-blocking-layer interfaces, with the former more pronounced (ss shown in figure 5). This ‘twin-peaks’ profile is characteristic of a p–i–n solar cell, with a layer of high electronic quality and low-doped semiconductor (intrinsic perovskite), between a p- and an n-layer. A question arises: how are free carriers generated in this system? Some scientists have suggested that, similarly to organic solar cells, PSCs can be considered as excitonic solar cells, which require a heterojunction interface to separate the photo-generated electron–hole pair. Some have suggested that, similarly to inorganic solar cells, the photo-excitations of perovskites spontaneously dissociate into free carriers in the bulk of the junction. Several reports have revealed a low binding energy for the exciton in perovskites (less than 50 meV) [35, 36]. The ultrafast interfacial charge-transfer dynamics [70] in combination with the observation of long electron- and hole-diffusion lengths points at PSCs being predominantly non-excitonic solar cells.

Alongside the above discussion, HTM-free PSCs have been under intense investigation. Etgar *et al* first reported on a hole-conductor-free CH_3_NH_3_PbI_3_/TiO_2_ heterojunction solar cell [71]. A thick perovskite film with large crystals, which function as light harvesters and hole transport materials at the same time, was deposited on top of mesoporous TiO_2_. In this case, a thick perovskite layer with a smooth surface is needed to avoid forming shunt pathways as well as to form good back contact to achieve high efficiency. The PV performance of the HTM-free PSC was dependent on the depletion layer width at the TiO_2_–CH_3_NH_3_PbI_3_ junction. This width can be enlarged by increasing the depleted fraction of TiO_2_, leading to a PCE of 10.85% [72]. A carbon counter-electrode used in a HTM-free PSC, collocated with a double layer of mesoporous TiO_2_ and ZrO_2_ as a scaffold, has been exploited by Mei *et al* [73]. The ZrO_2_ layer was found to block the flow of photogenerated electrons to the back contact, retarding recombination with the holes from the perovskite at the back contact. The cell achieved a certified PCE of 12.8%.

## 4. Progress in perovskite solar cell device performance

The first report of a solid-state PSC device was published in 2012 [21], in which CH_3_NH_3_PbI_3_ was deposited into a 0.6 *μ*m mesoscopic TiO_2_ film, covered with a hole-conductor spiro-OMeTAD. The device exhibited a large *J*_SC_ exceeding 17 mA cm^–2^, a *V*_OC_ of 0.888 V and a fill factor (FF) of 0.62, yielding a PCE of 9.7% under standard AM 1.5 sunlight. The use of spiro-OMeTAD dramatically improved the device’s stability compared to liquid junction cells in *ex situ* long-term stability tests conducted for over 500 h, where the devices were stored in air at room temperature without encapsulation. Subsequenty, this group has rarely reported the results of long-term stable PSC devices. After systematic optimization by modifying the conditions for the PbI_2_ precursor deposition and the transformation reaction, in 2013 Burschka *et al* reported PSC devices with PCEs up to 15% with *J*_SC,_ *V*_OC_ and FF values of 20.0 mA cm^−2^, 993 mV and 0.73, respectively [22]. Snaith and co-workers used a mixed halide system-CH_3_NH_3_PbI_3−*x*_Cl_*x*_ on both the TiO_2_ and Al_2_O_3_ mesoporous layers [65]. The highest efficiencies (PCE = 10.9%, *J*_SC_ = 17.8 mA cm^–2^, *V*_OC_ = 0.98 V and FF = 0.63) were obtained for the mesoporous Al_2_O_3_ devices where they acted purely as scaffolds and did not take part in the electrical processes. A much simpler approach involved the vapor deposition of perovskite films onto TiO_2_ films which yielded a short-circuit photocurrent of 21.5 mA cm^−2^, a *V*_OC_ of 1.07 V, a FF of 0.68 and an efficiency of 15.4% [23]. These concurrent reports sparked an explosion of research activity in which a variety of device configurations, deposition protocols and material sets have been employed. To establish systematic design rules to achieve high efficiencies in simple PSCs, we provide a review of deposition methods and device configuration optimization evolution routes in the following sections.

### 4.1. Preparation methods for highly efficient devices

#### 4.1.1. Solution process fabrication

**Figure 6.**
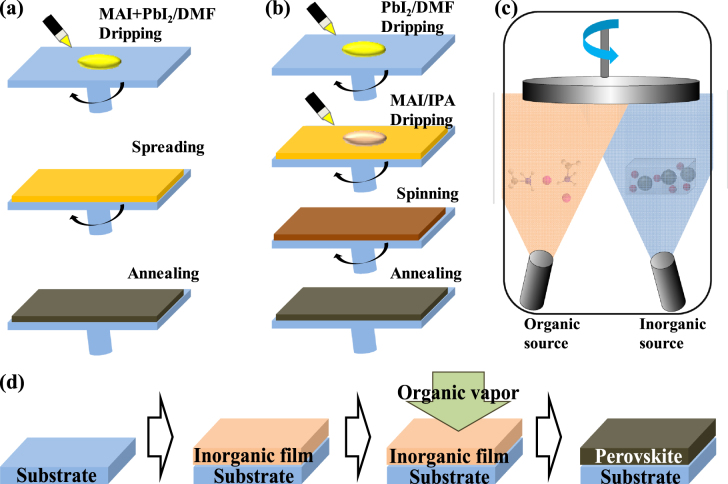
Four general methods for preparing perovskite active layers. (a) One-step precursor deposition, (b) two-step sequential deposition method, (c) DSVD and (d) VASP. Reprinted with permission from [66]. Copyright 2014 American Chemical Society.

To deposit CH_3_NH_3_PbI_3_ onto substrates, two methods have been developed, i.e., the so-called one-step or two-step coating methods (figures 6(a) and (b)). Perovskite thin film can be formed either by spin-coating a mixed CH_3_NH_3_I and PbI_2_ solution (one-step coating) or by spin-coating PbI_2_ followed by deposition of CH_3_NH_3_I (two-step coating). One-step precursor solution deposition represents the most popular thin film deposition method for PSCs due to its simplicity. Generally, the precursor solution is prepared by mixing powders of MAX (X: I, Br, Cl) and PbX_2_ (X: I, Br, Cl) at a 1:1 (stoichiometry) or 3:1 (non-stoichiometry) mole ratio in a polar aprotic solvent, such as N, N-dimethylformamide (DMF), gamma-butyrolactone (GBL), or dimethyl sulfoxide (DMSO). However, forming a homogeneous pin-hole-free perovskite layer is still a challenge for the one-step coating process. A poor coverage of the perovskite layer results in poor light absorption as well as a shunting path for charge recombination, which would greatly reduce the device’s efficiency [25, 60, 65]. Unlike polymeric films, which are mostly amorphous when deposited using the spin-coating technique, it is difficult to control the perovskite crystalline formation. Even a mild temperature heating treatment to remove solvent residues can result in dewetting and roughening of the as-deposited perovskite films [74].

By including additives in the precursor, a perovskite film with a smooth surface and uniform crystal domains can be formed as a compact layer for planar PSC devices. To improve the solubility of choline and the uniformity of the perovskite crystal layer, CH_3_NH_3_Cl [75], 1, 8-diiodooctane [76] or NH_4_Cl [77] have been added, resulting in PCEs of 12%, 11.8% and 9.93%, respectively. In addition, solvent engineering processes have been intensely investigated. A higher PCE was obtained when a mixture of DMF and GBL (97:3 in volume) was applied, compared with using GBL and DMF alone [78]. As an intermediate phase (CH_3_NH_3_I–PbI_2_–DMSO) was found when a mixture solvent of GBL and DMSO (7:3 in volume) was applied, the DMSO competed with CH_3_NH_3_I to retard the crystallization of perovskite [79]. After adding toluene to the solution drop-wise to remove the excess DMSO, a uniform intermediate layer was formed on the m-TiO_2_ and a certified PCE of 16.2% was achieved. Simultaneously, Xiao *et al* reported a similar study, in which immediately after spin-coating a CH_3_NH_3_PbI_3_ precursor DMF solution, chlorobenzene was added to induce the fast crystallization of perovskite [80]. A uniform CH_3_NH_3_PbI_3_ layer was formed, yielding an average PCE of 13.9 ± 0.7% on a compact TiO_2_ substrate. Likewise, the effects of post-annealing processes on perovskite film coverage and optoelectronic properties has also been characterized [54, 81, 82].

A significant development for solution-based deposition has been the application of the two-step sequential deposition method (originally developed by Mitzi *et al* [83, 84]), which was first used by Burschka *et al* to fabricate perovskite thin films in solar cell fabrication [22]. In a typical sequential deposition procedure, PbI_2_ is first spin-coated on a TiO_2_ layer from a solution under appropriate conditions (including solution concentration and spin coating speed) to enable full infiltration within the mesoporous layer. The yellow substrates are subsequently transformed into perovskite through dipping in a solution of MAI (MA: CH_3_NH_3_^+^ cations) in isopropanol. Perovskite prepared using the two-step coating method shows cuboid-like crystals, whereas the one-step method from the DMF solution of CH_3_NH_3_I and PbI_2_ produced a shapeless morphology [85, 86]. In PSCs with a mesoporous scaffold, the two-step sequential procedure allows much better control of the perovskite morphology compared to the one-step deposition method by allowing better confinement of PbI_2_ into the nanoporous network of TiO_2_. Due to the volume expansion [66] (∼75%) occurring during the conversion of PbI_2_ into CH_3_NH_3_PbI_3_, it can be expected that the mesoporous layer would be better infiltrated through the sequential deposition process. Thus, the two-step sequential deposition method provides a means to achieve excellent PV performance (∼15%) with high reproducibility [22].

#### 4.1.2. Vapor deposition method

Apart from the widely utilized solution-based deposition processes, Snaith and co-workers demonstrated efficient planar solar cells of CH_3_NH_3_PbI_3−*x*_Cl_*x*_ formed by dual-source evaporation of PbCl_2_ and CH_3_NH_3_I (figure 6(c)) [23]. In the dual-source evaporation procedure, CH_3_NH_3_I and PbCl_2_ are heated to about 120 °C and 325 °C, respectively, then deposited simultaneously onto c-TiO_2_ coated fluorine-doped tin oxide (FTO) glasses under a high vacuum. It was demonstrated that vapor-deposited perovskite films were extremely uniform with crystalline platelets at the nanometer scale. The authors claimed that the superior uniformity of the coated perovskite films without any pin-holes was the reason for the record efficiency of 15.4% [23]. Thermal evaporation requires a high vacuum, which restricts cost effectiveness and mass production. To overcome this problem, Yang and co-workers reported a novel low-temperature approach for the deposition of the perovskite absorbing layer called the vapor-assisted solution process (VASP, figure 6(d)), which grows perovskite films via *in situ* reactions of as-deposited films of PbI_2_ with CH_3_NH_3_I vapor [66]. The perovskite film derived from this approach exhibits full surface coverage, uniform grain structure with grain sizes up to micrometers and 100% precursor transformation completeness. A film evolution study of perovskite transformation indicated an appropriate rearrangement of PbI_2_ film during the intercalation of MAI driven by the reduction of grain boundary energy. Facilitated by the excellent film quality, the MAPbI_3_ materials enabled an impressive device PCE of 12.1% in a planar architecture.

The vacuum deposition method seems to present a simple and controllable approach in the pursuit of high-quality perovskite films compared to the solution process. Therefore, it is often used to investigate the effects of perovskite layer thickness on optoelectronic properties. Very recently, in an inverted planar perovskite device, the vacuum deposition method was used to fabricate CH_3_NH_3_PbI_3_ layers with different thicknesses to establish systematic design rules through cavity modeling and optimization of perovskite light-harvesting [35].

### 4.2. Interface engineering

**Figure 7.**
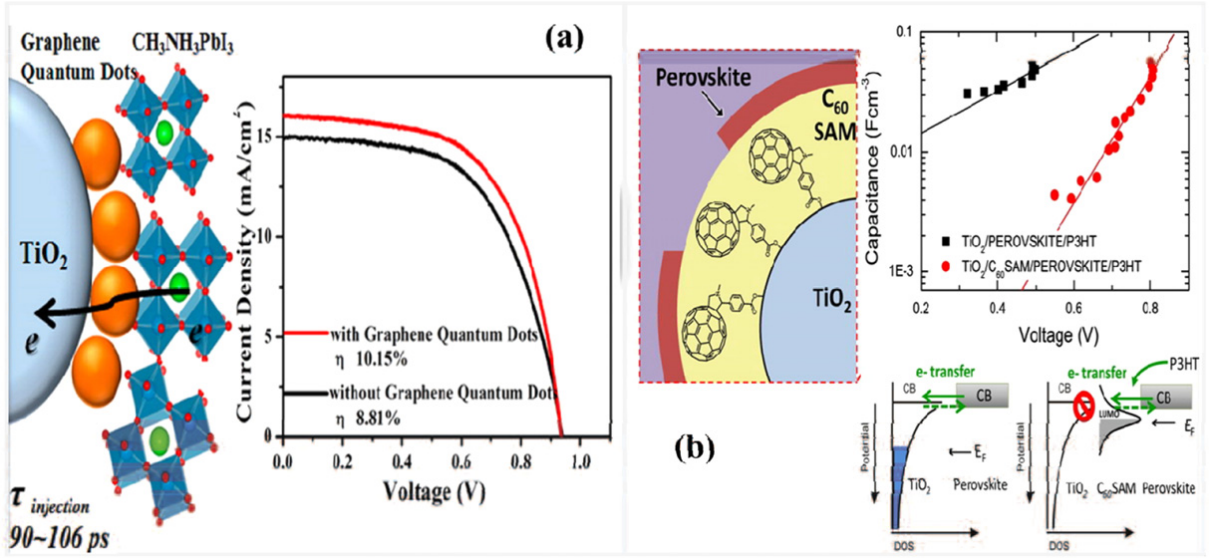
Surface engineering of high mobility materials to enhance the electronic coupling between perovskites and the n-type charge transport layer in mesoscopic nanostructure PSCs. Reprinted with permission from [87]. Copyright 2013 American Chemical Society. Also reprinted from [88]. Copyright 2014 American Chemical Society.

**Figure 8.**
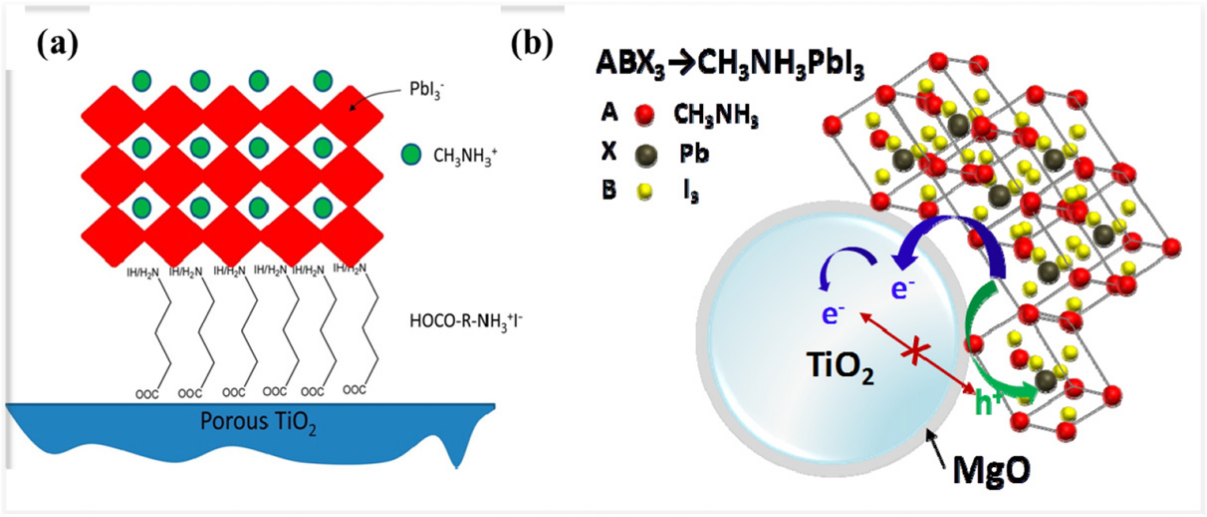
The TiO_2_ surface engineering works show retardation of charge recombination between perovskites and n-type charge transport layers, including (a) organic materials such as HOCO-R-NH^3+^I^−^ and (b) inorganic materials such as MgO. Part (a) reprinted with permission from [90]. Copyright 2014 American Chemical Society.

The interface engineering of mesoscopic materials has been demonstrated to be good way to control charge transport and recombination (figure 7). Abrusci *et al* reported a fullerene self-assembled monolayer (C_60_SAM) functionalized mesoporous TiO_2_ for PSCs [87]. The C_60_SAM acts as a very effective electron acceptor from the perovskite. Electron transfer from the perovskite to the TiO_2_ could be blocked and thus the device’s *V*_OC_ loss was reduced. This strategy allows the enhancement of the electronic coupling between perovskites and polymer semiconductors and hence represents an exciting route forward for PSCs. Similarly, Zhu *et al* inserted an ultrathin interlayer of graphene quantum dots (GQDs) between the perovskite and TiO_2_, and highlighted the beneficial role of GQDs in facilitating the electron transfer from the perovskite absorber to the collector, which could enhance PSC performance significantly [88]. Other studies proposed methods to prevent degradation, and enhance the stability and performance of PSCs, such as interface modification by inserting Sb_2_S_3_ or a HOCO-R-NH^3+^I^−^ layer on TiO_2_ (figure 8(a)) [89, 90]. Recently, an ultrathin MgO nanolayer was found to retard charge recombination between the electrons injected into TiO_2_ nanoparticles and the holes in perovskite materials (figure 8(b)) [91].

Zhou *et al* reported a new strategy of modifying indium tin oxide (ITO) with polyethyleneimine ethoxylate (PEIE) to reduce the work function and increasing the carrier density by doping yttrium into the TiO_2_ compact layer, thereby improving the electron transport channel in planar structure PSCs [54]. Zhang *et al* inserted polyelectrolyte intermediate layers such as PEIE and poly[3-(6-trimethylammoniumhexyl)thiophene] (P3TMAHT) and achieved a high efficiency of 12%, which was attributed to the formation of surface dipoles, thereby reducing the work function of the subsequently deposited metal [92].

**Figure 9.**
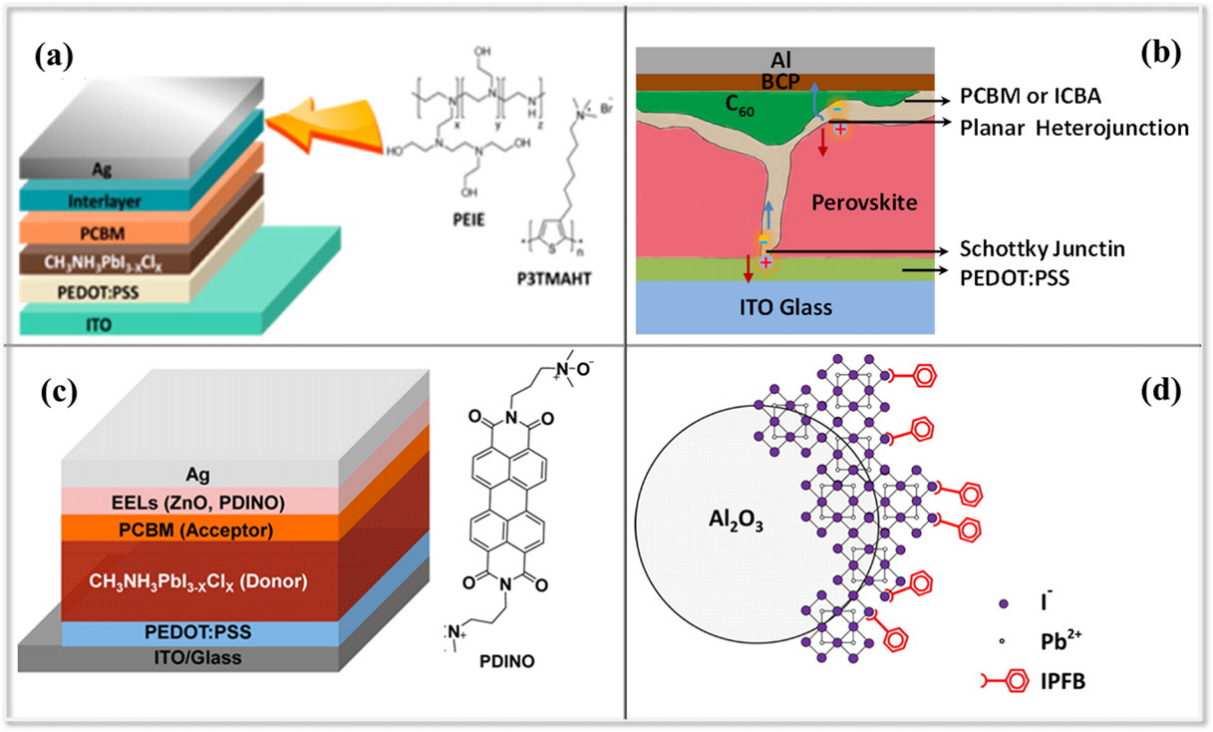
(a)–(c) Interface engineering between perovskite and metal electrode contact in planar nanostructure PSCs. The indene-C_60_ bisadduct (ICBA) or PCBM layer is shown as a conformal layer in (b) rather than as an electron extraction layer (EEL) as in (a) and (c). Reprinted with permission from [92]. Copyright 2014 American Chemical Society. Also reprinted from [96]. Copyright 2015 American Chemical Society. (d) Surface passivation of trap states at the perovskite surface. Reprinted with permission from [98]. Copyright 2014 American Chemical Society.

Similarly, interface engineering has attracted attention in the study of inverted planar structures because the extensive existing knowledge of hybrid interfaces and materials of the OPV community can be utilized (figures 9(a)–(c)). Wang *et al* and Xiao *et al* reported a novel electron selective layer design, which was a low-temperature solution process to form a continuous CH_3_NH_3_PbI_3_ layer and used a unique double fullerene layer ([6,6]-phenyl-C61-butyricacidmethyl (PCBM)/fullerene (C_60_)) structure to passivate the trap states, leading to a high efficiency inverted planar PSC (∼15.4%) with a record FF of 0.8 [93, 94]. As demonstrated in OPVs, the existence of an energy barrier between the organic material (PCBM) or perovskite semiconductor and top metal contact would significantly influence the built-in potential and charge carrier extraction in a p–i–n device structure [95]. Therefore, electron extraction interlayers such as n-type TiO_*x*_, ZnO nanocrystals and perylene diimide derivative (PDINO) were developed to align the energy levels between the perovskite semiconductor and top metal contact [55, 96, 97]. This is similar to the design of passivation of the trap states by preventing direct contact of the electrodes and the perovskite layers and thus enhancing the ambient stability of the devices at the same time, resulting in excellent ambient stability and PCEs as high as 10 ∼ 15.9%.

Taking this surface passivation strategy to the hole trapping states at the perovskite surface, which generate charge accumulation and consequent recombination losses, iodopentafluorobenzene (IPFB) was employed to cover the perovskite crystal and form a supramolecular halogen bond donor–acceptor complexation (figure 9(d)) [98]. Abate *et al* demonstrated that the uncoordinated halide ions on the surface of the organic–inorganic halide perovskite crystals would reduce the device’s performance via trapping positive charges at the interface of the perovskite/p-type hole-conductor heterojunction. Following this strategy, the ensuing solution processed PSCs exhibited a maximum PCE of 15.7%.

## 5. More exciting discoveries and open issues

### 5.1. Flexible and semitransparent perovskite solar cells

PSCs are fairly attractive as flexible solar cells due to their low-temperature processing (below ∼150 °C), the all-solid-state nature of the thin films and their high efficiency. Since the conventional process for the fabrication of electron transporting materials (ETMs), such as a TiO_2_ compact layer, required a high temperature (over ∼400 °C), most flexible PSCs on ITO-coated poly(ethyleneterephthalate) (PET) substrates have adopted the inverted planar structures of poly(3,4-ethylenedioxythiophene)poly(styrenesulfonate) (PEDOT:PSS)/perovskite/ PCBM architectures [55, 99, 100]. Carmona *et al* annealed the polymer conductor (PEDOT:PSS) on an AZO/Ag/AZO coated PET flexible substrate and obtained a PCE of 7% based on the CH_3_NH_3_PbI_3_ perovskite absorber [100]. Fexible PSCs composed of regular structures with low-temperature deposited ZnO ETM, titanium (Ti) foil/TiO_2_ nanotubes, or atomic layer deposited TiO_*x*_ have been reported [58, 101, 102]. These solar cells shows superior mechanical endurance properties and PCEs ranging from 8–12%, demonstrating that flexible PSCs are potentially usable as a power solution for future wearable devices.

Semitransparent solar cells are commercially desirable for integrating solar cells into the windows and cladding of buildings and automotive applications. Eperon *et al* first reported the morphological control of perovskite thin films to form semitransparent planar heterojunction solar cells with a neutral color and comparatively high efficiencies (PCE ∼8% at 30% average visible transmittance) [103]. Recently, semitransparent PSCs with high PCEs (∼10–13%) at an average transmittance of the full devices in the range 7–16% were developed with solution processed ultra-thin perovskite layer and highly transparent and conductive top electrodes [104]. Integrated with flexible substrates, this technology now enables PSCs to not only compete for high-efficiency opaque applications but also offer an ideal solution to building integrated PVs.

### 5.2. Perovskite solar cells with high output voltage

As discussed above, different halide elements will change the position of the CB and VB of perovskites. As a result, a device with a high output photovoltage could potentially be obtained. CH_3_NH_3_PbBr_3_-based PV devices with poly[N-9-hepta-decanyl-2,7-carbazole-alt-3,6-bis(thiophen-5-yl)-2,5-dioctyl-2,5-di-hydro-pyrrolo[3,4-]pyrrole-1,4-dione] (PCBTDPP) as the HTM displayed a considerably high *V*_OC_ of ∼1.15 V, which was suggested to be due to several factors, including the negligible difference between the VB maximum of CH_3_NH_3_PbBr_3_ and the highest occupied molecular orbital (HOMO) level of PCBTDPP, the large offset between the CB minimum of CH_3_NH_3_PbBr_3_ and the quasi-Fermi levels of TiO_2_, and the good interaction between TiO_2_/perovskite/PCBTDPP, as well as the superior hole mobility of PCBTDPP [46]. Subsequently, Ryu *et al* further reduced the HOMO of the HTM using a series of triarylamine polymer derivatives such as indenofluorene (PIF8-TAA). The resultant CH_3_NH_3_PbBr_3_ PSC showed an improved *V*_OC_ to 1.4 V with a FF of 0.79 [105]. Considering hole-conductor-free PSCs, an Al_2_O_3_/CH_3_NH_3_PbBr_3_ PSC with a high *V*_OC_ of 1.35 V was demonstrated, showing a major effect from the perovskite/metal oxide interface on the output voltage [106]. Edri *et al* achieved a record *V*_OC_ of 1.5 V with a CH_3_NH_3_PbBr_3−*x*_Cl_*x*_-based PSC based on a organic–inorganic lead bromide and 4, 4’-bis(N-carbazolyl)-1,1’-biphenyl (CBP) hybrid structure by replacing fractional Br by Cl [107]. These high output voltage devices have shown promising application prospects for tandem PVs.

### 5.3. Hysteresis and stability

The appearance of hysteresis in the photocurrent–voltage curves—which consists of the anomalous dependence of the PCE on the voltage scan direction and speed—calls our attention to device stability and the pitfalls of PCE measurements. This hysteresis been tentatively attributed to the paraelectric or even ferroelectric properties of perovskites at room temperature and above [108, 109]. Simulations suggest that the internal electrical fields associated with microscopic polarization domains contribute to hysteretic anomalies in the current–voltage response of PSCs due to variations in electron–hole recombination in the bulk [110]. However, others would suggest that, due to its low lattice energy, organometal halide perovskite tends to possess strong ionic characteristics, which are sensitive to polarization in an electric field [111]. An impedance study has shown that a high value of the dielectric constant at low frequencies results from a combination of dipolar, ionic and electronic contributions, which are the main reasons for the *J*/*V* hysteresis [112–115]. although experimental evidence for the proposed causes of hysteresis in PSCs is lacking.

The stability of PSCs is another issue. The negative standard Gibbs free energy for iodide perovskite degradation with moisture has indicated the sensitivity of perovskite to water [116]. An understanding of the degradation mechanism will be helpful in enhancing long-term stability, which will be a significant criterion for the commercialization of PSCs. However, very few stability studies have been performed so far [22, 73, 117] and most of them cannot be repeated by others. At this stage it is still unclear whether PSCs can meet the stringent international norms for outdoor PV applications.

## 6. Summary and outlook

The recent progress in hybrid lead halide PSCs is remarkable. New reports on working mechanisms, device structures, preparation methods and applied materials continue to emerge. We have given an overview of the optoelectronic properties of organometal halide perovskite materials and revisited the optimization routines in different architectures, including perovskite deposition processes and interlayer engineering. In order to reveal the working principles and degeneration processes in PSC devices, not only photoluminescence and impedance analyses, which have been described as important tools to characterize the charge separation, carrier transport, recombination and diffusion length in PSCs, DSSCs and OPVs, but also novel characterization methods need to be introduced to understand these types of PV devices [69, 118]. The fabrication methods were shown to influence crystal growth and the morphologies of perovskite materials, thereby affecting the resultant PSC performance. The recent progress underlying solution engineering exhibits the ability of controlling the surface morphology of perovskite films.

In the case of MAPbI_3_ perovskite with a bandgap of 1.57 eV (corresponding to an onset of light absorption of ∼790 nm), a short circuit current density (*J*_SC_) of 28 mA cm^−2^ is theoretically achievable. To date, by optimizing the formation of the perovskite layer with Cl doping, a *J*_SC_ of 22.7 mA cm^−2^ and a FF of 0.75 have been achieved. In addition to further optimizing the morphology of the perovskite absorber (thickness and homogeneity), we should look into finding better selective interlayer materials with higher mobility, and possibly interface engineering, to balance charge transport and minimize recombination.

At present the PSC field is still in a dynamic state. Reports on further improvements in performance are expected in the near future, where reaching a PCE of 25% now seems to be a realistic goal. Furthermore, PSCs are attractive candidates for the top cell in a two-level tandem configuration, which employs crystalline silicon or copper indium gallium selenide in the bottom cell. The high-efficiency and cost-effective materials and processes for PSCs make them economically viable for commercialization. However, large-scale deployment of perovskite cells will depend on whether the stability and toxicity issues can be solved. Technological developments in the areas of nontoxic and stable scalable manufacturing techniques need to be effectively addressed.
